# *PRDM16* Gene Polymorphism Is Associated with Obesity and Blood Lipids Profiles in Saudi Population

**DOI:** 10.3390/jcm7060141

**Published:** 2018-06-08

**Authors:** Aishah AlAmrani, Mouaadh AbdelKarim, Mohammed AlZoghaibi

**Affiliations:** 1 Department of Physiology, Faculty of Medicine, King Saud University, Riyadh 11461, Saudi Arabia; malzoghaibi@ksu.edu.sa; 2 Faculty of Applied Medical Sciences, Tabuk University, Tabuk 47914, Saudi Arabia; 3 Department of Physiopathology of Inflammatory Bone Diseases, University of the Littoral, Opal Coast, F-62327 Boulogne sur Mer, France; mouaadh.abdelkarim@univ-littoral.fr

**Keywords:** *PDRM16*, *PDE4D*, obesity, blood lipids, Saudi population

## Abstract

Aims: The PR domain containing 16 (*PRDM16*) gene and the Phosphodiesterase 4D (*PDE4*) gene are both an essential regulators in the thermogenesis process in the brown adipose tissues (BAT). The influence of polymorphisms in those genes on obesity and blood lipids profile is unknown particularly in the Saudi population, so the current study is aiming to explore that. Methods: A case control format was used that involved 89 obese individual and 84 non-obese (control). The *PRDM16* (*rs2651899*) and *PDE4D* (*rs295978*) polymorphisms were genotyped using KASP™ (Competitive Allele-Specific PCR) method. Results: The distributions of the AA, GG, and AG genotypes of *PRDM16* (*rs2651899*) polymorphism were 0.19, 0.26 and 0.54, respectively. While the distribution of the mutated allele A was 0.7 in the obese group comparing to 0.34 in the non-obese group. Participants with the mutated genotypes, AA and AG, of *PRDM16* (*rs2651899*) polymorphism were significantly more likely to be obese as compared to participants with wild type genotype (OR = 21, 95% CI = 5.4190 to 84.4231, *p* value < 0.0001 and OR = 44.6, 95% CI = 11.5984 to 172.0157, *p* value < 0.0001, respectively). The wild type GG genotype of this polymorphism was associated with higher blood cholesterol, HDL and LDL but lower blood triglyceride compared with the mutated genotypes (*p* = 0.003, *p* = 0.008, *p* = 0.02 and *p* = 0.003, respectively). In contrast, *PDE4D* (*rs295978*) polymorphism was not associated with risk of obesity and had no effects on blood lipids profile. Conclusions: We found that the PRDM16 polymorphism (*rs2651899*) is a risk factor for obesity and influence blood lipids profiles significantly in Saudi population. While the *PDE4D* (*rs295978*) polymorphism didn’t show significant effect on risk of obesity or blood lipids profiles.

## 1. Introduction

Obesity is considered the fifth highest cause of death worldwide by the World Health Organization [1]. In Saudi Arabia, 29% of the population is affected by obesity compared with 13% worldwide [2]. Obesity poses a substantial health risk, and contributes to a range of serious health problems, including atherosclerotic and cerebrovascular disease, several types of cancer, blood dyslipidaemia, hypertension, gallbladder disease, and diabetes mellitus. Despite extensive research, the molecular mechanisms behind obesity pathophysiology remain largely undefined. However, recent evidence suggests that the thermogenesis process is impaired in brown adipose tissues (BAT), contributing to obesity pathogenesis.

Moreover, BAT is a distinctive type of adipose tissue that disperses energy through nonshivering thermogenesis. This process is mediated by the uncoupling protein 1 (*UCP1*), which is abundant in that tissue’s inner mitochondrial membrane [3]. Earlier studies indicated the central role of BAT in energy haemostasis and in preventing obesity in mammalians, while it has been demonstrated that BAT activation resists obesity and metabolic disorders in animal models [4,5,6,7,8]. Recently, it has been shown that obesity is associated with a reduction or impairment of BAT activities in classical BAT areas, detectable by radiological images (Positron Emission Tomography-Computed Tomography (PET/CT)) [9,10]. However, the altered molecular mechanisms associated with reduced BAT activities in obesity have not been characterised in humans.

The PR domain containing 16 (*PRDM16*) gene and the Phosphodiesterase 4D (*PDE4*) gene are both essential regulators in the thermogenesis process in BAT. *PRDM16* is a transcriptional coregulatory that induces the formation of BAT in classic BAT areas [11], as well as the BAT-like cells in white adipose tissues [12]. Interestingly, the removal of *PRDM16* in mice has been found to lead to a loss of BAT functions and lower *UCP1* and PGC1α (the BAT transcriptional inducer) expression [13]. Moreover, phosphodiesterase 4D (*PDE4D*) is an enzyme belonging to the phosphodiesterase enzymes family that accelerates the hydrolysis of cAMP [14]. As cAMP is an essential molecule in the BAT thermogenesis process, *PDE4D* has been found to inhibit thermogenesis in BAT. Interestingly, *PDE4D* has also been found to be a regulator of *UCP1* gene expression and lipolysis, while the inhibition of *PDE4D* induces the expression of *UCP1* and lipolysis [15]. The effect of *PDE4D* on the action of *PRDM16* gene was not investigated directly earlier, but it seems that the *PDE4D* may reduce the *PRDM16* function in BAT through reducing the bioavailability of cAMP, this required further studies.

The *PRDM16* polymorphism was found to be associated with obesity among Chinese males [16] and to lean body mass among Japanese women [17]. In addition, an association has been found between *PRDM16/rs2651899* and migraines among different populations [18,19]. No prior studies have assessed the association of *PDRM16*/*rs2651899* with obesity or blood lipids profile, particularly among Saudi populations. Further, no earlier study has assessed the association of *PDE4D* polymorphisms with either obesity or lipids profiles.

In the current study, we have assessed the association between *PDRM16/rs2651899* and *PDE4D/rs295978* polymorphisms with obesity and blood lipids profiles among the Saudi population.

## 2. Methods and Materials

### 2.1. Participant Recruitment

This case control study recruited 89 obese and 84 non-obese healthy individuals, all of whom were visitors to King Khaled University Hospital (KKUH), Riyadh City, Saudi Arabia. The participants were Saudi adults with a BMI greater than 30 for the obese group and less than 30 for the non-obese group. The participants were accepted if they had a current health problem. Ethical approval of the study protocol was received from the Institutional Review Board (IRB) of the College of Medicine, King Saud University (Ref. No. 16/0283/IRB). The researchers obtained informed consent, as approved by the IRB, from the participants. Weight, height and blood pressure were measured using medical scales available at KKUH.

Moreover, the sample size was determined according to the method described earlier [20], with a confidence level of 95%, power of 85% and the critical value is 1.96

### 2.2. Sample Collection

About 5 mL of venous blood was extracted, with 2 mL placed in an ethylenediaminetetraacetic acid (EDTA) tube and 3 mL placed in a gel separator tube. The EDTA tubes were stored at 4 °C until used for DNA extraction, and the gel separator tubes were centrifuged to obtain serum. The serum was allocated and stored at −80 °C until use in biochemical analysis.

### 2.3. DNA Extraction

The DNA was extracted from EDTA tubes using TRIGent™ (CAT. No. K5161, Biomatik, Wilmington, DE, USA), a Trizol equivalent, according to the manufacturer’s instructions. The DNA quality and quantity were measured using NanoDrop ND-1000 UV-VIS Spectrophotometer version 3.2.1 (Thermo Fisher Scientific, Waltham, MA, USA). The DNA was then stored at −80 °C until it was used for genotyping.

### 2.4. SNP Genotyping

The KASP™ Competitive Allele-Specific PCR method (developed by Kbioscience, Hoddesdon, UK) was used for genotyping selected SNPs following the manufacturer’s instructions. The reaction was run in a real-time PCR machine (The Applied Biosystems™ ViiA™ 7 system, Applied Biosystems, Foster City, CA, USA). The ensuing signal was read by the same PCR machine, and the genotypes were called automatically by the real-time PCR machine.

### 2.5. Blood Lipid Profile

Lipids profiles were measured using Dimension Vista™ 1500 System available in the KKUH laboratory department. The obtained serum from the venous blood samples was thawed at room temperature then about 0.1 mL used for each blood lipids assays as recommended by the manufacture. 

### 2.6. Statistical Analysis

The data were normally distributed as found by Anderson Darling normality test (*p* > 0.05). The data were presented as mean and standard deviation. The frequency of each genotype was compared in the obese vs. non-obese groups. The association of each genotype with obesity and blood lipids profile were calculated using odds ratio (OR). The difference between the groups was assessed using analysis of variance (ANOVA) test followed by post-hoc Tukey honest significant difference (HSD) test. The result was considered significant if the *p* value was less than 0.05. The Statistical Package for the Social Sciences (SPSS) program version 20 (IBM, New York, NY, USA) was used. The statistical power of estimating genetic correlation was 0.05 (standard error 1.9), calculated using the method described earlier [21].

## 3. Results

### 3.1. Characteristics of the Study Participants

The characteristics of the study participants are presented in Table 1. A total of 173 participants were involved in the study. Eighty-nine were obese (BMI > 30 kg/m^2^) and 84 were non-obese (BMI < 30 kg/m^2^). The mean BMI was 35 kg/m^2^ (±5.5) in the obese group and 25 kg/m^2^ (±3) in the non-obese group (*p* < 0.0001). The two groups were comparable in age and sex (*p* = 0.3, *p* = 1.0, respectively). Interestingly, we found that the non-obese participants had a higher high-density lipoprotein cholesterol (HDL) level compared with the obese group (1.3 mmol/L vs. 1.1 mmol/L, *p* = 0.0009), while there was no significant difference in blood cholesterol, low-density lipoprotein cholesterol (LDL), or blood triglyceride levels among the two groups (*p* = 0.5 and *p* = 0.1, respectively). We found that obese participants had higher systolic blood pressure compared to non-obese (122 mmHg vs. 115 mmHg, respectively, *p* = 0.003). There was no significant difference in diastolic blood pressure (*p* = 1.0).

### 3.2. Genotype Frequencies and Distribution

**Figure 1:**
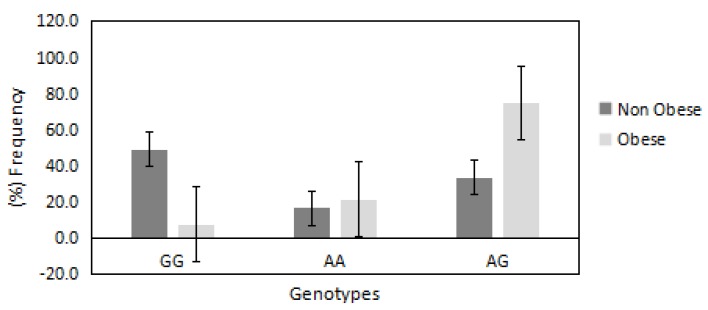
Distribution of *PRDM16/rs2651899* genotypes in obese and non-obese groups. The frequency of the mutated genotypes AA and AG was higher in obese compared with non-obese participants (75% vs. 33% and 21% vs. 16%, respectively). In contrast, the frequency of wild-type GG genotype found to be very low in obese compared with non-obese participants (7% vs. 49%).

The distributions of AA, GG and AG genotypes of *PRDM16/rs2651899* polymorphism were 0.19, 0.26 and 0.54, respectively. This distribution was not deviated significantly from the Hardy Weinberg equilibrium (*p* = 0.09). The chi-square random variable (*χ*^2^) of the Fisher exact test was 53.0 (*p* < 0.00001), indicating the high dependence of participants’ BMI on *PRDM16/rs2651899* genotypes. Figure 1 shows the distribution of *PRDM16/rs2651899* genotypes in obese compared with non-obese participants. The frequencies of mutated genotypes (AA and AG) were higher in the obese compared with the non-obese (75% vs. 33% and 21% vs. 16%, respectively), suggesting the causative effect of this genotype on obesity. In contrast, the frequency of wild-type GG genotype was found to be lower in the obese compared with the non-obese group (7.9% vs. 49%).

**Figure 2:**
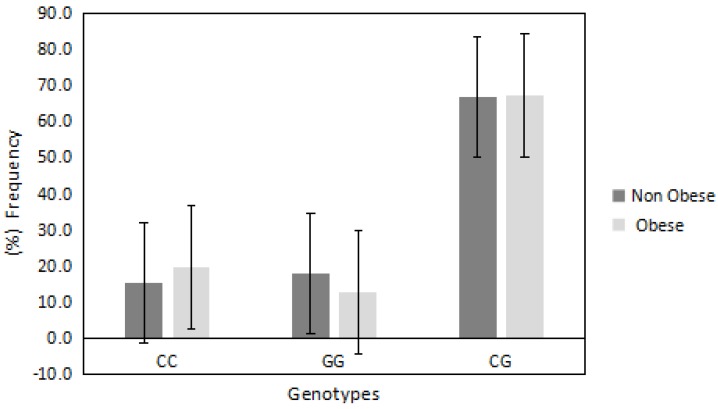
The frequency of *PDE4D/rs295978* genotypes in obese and non-obese groups. The heterozygote mutated CG genotype was semi-equal in the obese and non-obese groups. The wild-type CC genotype was relatively higher in the obese group compared with the non-obese group, while the mutated GG genotype was lower in the obese group compared with the non-obese group.

In regards to *PDE4D/rs295978* polymorphism, the distributions of CC, GG and CG were 0.178, 0.15 and 0.67, respectively. This deviated from the Hardy Weinberg equilibrium (*p* = 0.0006). The chi-square random variable (*χ*^2^) of the Fisher exact test was 1.2 (*p* = 0.5), indicating the independence of participants’ BMI from the *PDE4D/rs295978* polymorphism. Figure 2 shows the frequencies of different genotypes among obese vs. non-obese participants. The mutated heterozygote CG genotype was the highest among all other genotypes, however, the frequency of this genotype was semi-equal in obese and non-obese groups (67% vs. 66%, respectively). The CC genotype was relatively higher in obese compared with non-obese (19% vs. 15%, respectively) while the GG genotype was lower in obese compared with non-obese (12% vs. 17%, respectively).

### 3.3. Association with Risk of Obesity

Table 2 demonstrates the association of *PRDM16/rs2651899* and *PDE4D/rs295978* polymorphisms with a risk of obesity. The mutated genotypes (AA and AG) of *PRDM16/rs2651899* polymorphism were associated with high ODD ratio compared with the wild-type GG genotype (ODD = 8, 95% CI = 2.8308 to 22.7266, *p* = 0.0001 and ODD = 13, 95% CI = 5.6623 to 33.8973, *p* < 0.0001, respectively), suggesting that these genotypes increase the risk of obesity. In regards to *PDE4D/rs295978* polymorphism, the mutated genotypes (GG and CG) were associated with reduced ODD of obesity but was statistically insignificant (OR = 0.5, 95% CI = 0.1969 to 1.5767, *p* = 0.2 and OR = 0.7, 95% CI = 0.3520 to 1.7490, *p* = 0.5, respectively).

### 3.4. Association with BMI and Blood Lipids Profile

Table 3 presents the difference in BMI, blood lipids and blood pressures among the *PRDM16/rs2651899* and *PDE4D/rs295978* genotypes groups. There was a significant difference in BMI and blood lipids profile among *PRDM16/rs2651899* genotypes groups. Participants with mutated genotypes (AA) and (AG) demonstrated significantly higher BMI (*p* = 0.001) compared with participants with GG genotype. Unexpectedly the wild type genotype of *PRDM16/rs2651899* polymorphism (GG) was associated with higher blood cholesterol, HDL and LDL but lower blood triglyceride compared with the mutated genotypes (*p* = 0.003, *p* = 0.008, *p* = 0.02 and *p* = 0.003, respectively), suggested the important of *PRDM16* in blood lipids regulation. Among the *PDE4D* polymorphism (*rs295978*) genotype groups, no significant difference in BMI or blood lipids was found except for HDL, which was higher in participants with mutated genotype GG (*p* = 0.05) compared with participants with wild-type CC genotype. Further, there were no significant differences in blood lipids or blood pressure levels among these groups.

## 4. Discussion

The current study assessed the association of *PRDM16* gene polymorphism (*rs2651899*) and *PDE4D* gene polymorphism (*rs295978*) with obesity and blood lipids profiles in the Saudi population. We found that *PRDM16* gene polymorphism (*rs2651899*) was associated with a significantly increased risk of obesity and a significant effect on blood lipids profile. In contrast, *PDE4D* polymorphism (*rs295978*) was not associated with a risk of obesity and had no significant effects on blood lipids profile except for HDL, which was higher in participants with the mutated genotype (GG) of this polymorphism (*p* = 0.05).

Our findings support the appreciated role of *PRDM16* in the BAT thermogenesis process. *PRDM16* highly expressed in BAT is also found to induce brown phenotype in WAT, which is found to directly activate PGC1α (BAT transcriptional inducer) and enhance the thermogenesis process ([22]). *PRDM16* polymorphism (*rs2651899*) is an intron variant that could affect the *PRDM16* gene splicing or downstream regulatory elements, which can lead to an altered expression of *PRDM16* mRNA. Our study did not shed light on the effect of this polymorphism on the expression of the *PRDM16* gene. However, the increased OR of obesity with the mutated genotypes of this polymorphism (AA and AG) suggests that this polymorphism significantly reduces the expression of the *PRDM16* gene, thus leading to a lower thermogenesis process and increased BMI.

The association between the *PRDM16* polymorphism and the blood lipids profile reflects the importance of this gene (as well as BAT) in lipid metabolism in humans. In the current study, the mutated genotypes (AA and AG) were found to be associated with lower cholesterol, LDL and HDL levels. In contrast, the wild-type GG genotype was associated with a lower triglyceride level.

However, it has been demonstrated in mice that BAT activation reduce plasma triglyceride by increasing its uptake into the BAT [22,23]. In agreement with that we found that the triglyceride level in patients with wild-type genotype of *PRDM16* polymorphism (*rs2651899*) was reduced. This could be explained by the fact that the intact expression of *PRDM16* gene in participants with wild-type genotype enhances the thermogenesis process, which leads to an increased uptake of blood triglyceride by BAT.

Moreover, it has been well established that BAT activation decreases plasma triglycerides in mice, but its effects on blood cholesterol and other blood lipids are still in debate in animal models and in humans. Recently, Dong et al. (2013) demonstrated that BAT activation by cold or adrenergic stimulation aggravated dyslipidemia by increasing plasma levels of cholesterol, LDL cholesterol, and glycerol up to 2- to 3-fold in mice [24]. Another study also showed that BAT activation aggravates hypercholesterolemia and atherosclerosis in Apoe^−/−^ and Ldlr^−/−^ mice [15]. In agreement, our study also demonstrated that the wild-type of *PRDM16* polymorphism (*rs2651899*) is associated with increased plasma level of cholesterol, LDL and HDL. These results suggest that enhanced BAT activities in participants with this genotype lead to increased plasma level in cholesterol, LDL and HDL. It is possible that the study participants are living obesogenic lifestyles (common among the Saudi population) so their bodies are compensating by increasing BAT activities, leading to increased plasma lipids. These findings support the hypothesis that BAT activation could aggravate dyslipidemia in humans. Therefore, it is of great importance that we find a mechanism to overcome BAT-induced dyslipidemia when considering BAT activation as treatment for obesity.

In regards to *PDE4D* polymorphism (*rs295978*), the distributions of CC, GG and CG were 0.178, 0.15 and 0.67, respectively. The mean BMI among participants with wild-type CC genotype of this polymorphism was relatively high (32.8 ± 8 kg/m^2^) compared with participants with mutated genotypes (GG and CG genotypes, 29.3 and 30.8 kg/m^2^, respectively). However, the difference in BMI remained insignificant; this may be due to the low sample size or because there is no gap in BMI between the obese and non-obese group.

Although there was an insignificant difference in BMI among *PDE4D* polymorphism (*rs295978*) genotypes groups, our data supported to a lesser extent the hypothesis that the *PDE4D* enzyme reduces the body’s thermogenesis process, thus increasing BMI. Although our data is suggestive and no investigation has been conducted on the underlying mechanisms, we propose that this mutation reduces transcription of the *PDE4D* gene. In turn, the bioavailability of cAMP increases and the thermogenesis process is enhanced; therefore, participants with mutated genotypes of this polymorphism demonstrate lower BMI.

Moreover, as mentioned earlier, the major disadvantage of BAT activation is an increase in the lipolysis process, which leads to an increase in plasma lipids. Our study also demonstrated that the mutated genotype (GG) of *PDE4D* polymorphism (*rs295978*) is associated with an increased blood HDL level. This suggests that the mutated genotype attenuates *PDE4D* enzyme function in inhibiting BAT activities; in turn, this can lead to an increased thermogenesis process, which leads to increases in blood HDL level. In support of this hypothesis, it has been demonstrated that BAT activation by cold exposure increased plasma HDL in humans [25]. Further studies are required to enhance our understanding of the role of BAT activities in regulating blood lipids.

It is noteworthy that our study has several limitations: small sample size, lack of randomisation, and the BMI difference between the obese and the non-obese group is not wide enough. These factors could explain why the effect of *PDE4D* polymorphism (*rs295978*) on BMI or blood lipids was insignificant. In addition, we did not examine the effects of the studied mutations on gene expression or thermogenesis process. We recommend repeating this assessment using a larger sample size to confirm findings in different populations.

In conclusion, we found that the *PRDM16* polymorphism (*rs2651899*) is a risk factor for obesity and significantly influences plasma lipids in the Saudi population. In our study, *PDE4D* polymorphism (*rs295978*) did not show a significant effect on BMI or blood lipids.

## Figures and Tables

**Table 1:** Characteristics of the study participants.

	Total (173)	Obese (89)	Non-obese (84)	*p* Value
Age (years)	32 (±10)	35.6 (±10)	32.4 (±9)	0.3
Male %	20.5%	20%	21%	1.0
BMI (kg/m^2^)	29.5 (±7)	35.5 (±5.5)	25.3 (±3)	<0.0001
Cholesterol (mmol/L)	4.5 (±0.9)	4.4 (±0.9)	4.5 (±1.1)	0.5
HDL (mmol/L)	1.2 (±0.3)	1.1 (±0.28)	1.3 (±0.4)	0.0009
Triglyceride (mmol/L)	1.2 (±0.7)	1.28 (±0.6)	1.1 (±0.9)	0.1
LDL (mmol/L)	3.1 (±0.8)	3.1 (±0.9)	3.1 (±0.8)	0.15
Systolic BP (mmHg)	88 (±14)	122 (±15)	115 (±12)	0.003
Diastolic BP (mmHg)	71 (±12)	71 (±9)	71 (±13)	1.0

There was a significant difference in BMI, HDL, and systolic blood pressure between the obese and the obese groups (*p* < 0.0001, *p* = 0.0009 and *p* = 0.003, respectively). BMI = Body mass index, HDL = high density lipoprotein cholesterol, LDL = low density lipoprotein cholesterol, BP = blood pressure. Bold values are statistically significant at *p* < 0.05.

**Table 2:** Association of *PRDM16/rs2651899* and *PDE4D/rs295978* genotypes and alleles with risk of obesity.

***PRDM16/rs2651899***					
**Genotypes**	**(GG)**	**(AA)**	**(AG)**	**Allele (G)**	**Allele (A)**
Obese (%)	18	35	67	30	70
Non-obese (%)	82	65	24	66	34
OR	1	8.0	13.0	1	4.5
95% CI	Reference	2.8308–22.7266	5.6623–33.8973	Reference	2.4980–8.2127
*p* value	—	*p* = 0.0001 *	*p* < 0.0001 *	—	*p* < 0.0001 *
***PDE4D/rs295978***					
**Genotypes**	**(CC)**	**(GG)**	**(CG)**	**Allele (C)**	**Allele (G)**
Obese (%)	62.5	48.2	56.7	51	49
Non-obese (%)	37.5	51.8	43.3	47	52
OR	1	0.5	0.7	1	1.5
95% CI	Reference	0.1969–1.5767	0.3520–1.7490	Reference	0.6603–2.0083
*p* value	—	*p* = 0.2	*p* = 0.5	—	0.6

The mutated genotypes AA and AG of *PRDM16/rs2651899* polymorphism were associated with high ODD ratio. While there was insignificant effect of *PDE4D/rs295978* genotypes on risk of obesity (*p* > 0.05). OR = ODD ratio, CI = confidence interval. * Statistically significant at *p* value < 0.05.

**Table 3:** Difference in BMI, blood lipids profile and blood pressures among the *PRDM16/rs2651899* and *PDE4D/rs295978* genotypes groups.

***PRDM16/rs2651899***							
	**Genotypes**			**ANOVA**	**Tukey HSD**		
	**GG** **MN (±SD)**	**AA** **MN (±SD)**	**AG** **MN (±SD)**	**F (*p*)**	**GG vs. AA** **Q (*p*)**	**GG vs. AG** **Q (*p*)**	**AA vs. AG** **Q (*p*)**
BMI (kg/m^2^)	25 (±3)	32 (±8)	33 (±6)	20 (<0.00001) *	6.6 (0.001) *	0.7 (0.8)	8.9 (0.001) *
Cholesterol (mmol/L)	4.6 (±0.7)	3.8 (±0.8)	4.6 (±0.9)	5.8 (0.003) *	3.9 (0.01) *	4.6 (0.003) *	0.1 (0.8)
HDL (mmol/L)	1.3 (±0.3)	1.0 (±0.3)	1.1 (±0.3)	4.9 (0.008) *	4.2 (0.008) *	1.7 (0.4)	3.3 (0.05) *
LDL (mmol/L)	3.0 (±0.6)	2.5 (±0.8)	3.2 (±0.4)	3.8 (0.02) *	2.8 (0.1)	3.8 (0.01) *	0.8 (0.8)
Triglycerides (mmol/L)	0.9 (±0.3)	1.0 (±0.3)	1.4 (±0.4)	5.8 (0.003) *	1.0 (0.7)	4.5 (0.004) *	3.0 (0.08)
SBP (mmHg)	119 (±10)	117 (±9)	120 (±11)	0.5 (0.5)	0.5 (0.8)	0.2 (0.8)	1.4 (0.5)
DBP (mmHg)	70 (±10)	71 (±9)	69 (±10)	0.2 (0.5)	0.2 (0.8)	0.7 (0.8)	0.8 (0.8)
***PDE4D/rs295978***							
	**Genotypes**			**ANOVA**	**Tukey HSD**		
	**CC** **MN (±SD)**	**GG** **MN (±SD)**	**CG** **MN (±SD)**	**F (*p*)**	**CC vs. GG** **Q (*p*)**	**CC vs. CG** **Q (*p*)**	**GG vs. CG** **Q (*p*)**
BMI (kg/m^2^)	32 (±8)	29 (±6)	30 (±7)	1.0 (0.3)	2.1 (0.3)	1.3 (0.6)	1.3 (0.6)
Cholesterol (mmol/L)	4.1 (±1)	4.6 (±1.1)	4.6 (±0.9)	2.0 (0.1)	2.1 (0.2)	2.0 (0.1)	0.1 (0.8)
HDL (mmol/L)	1.0 (±0.3)	1.2 (±0.3)	1.0 (±0.2)	3.3 (0.03) *	3.2 (0.05) *	0.2 (0.8)	3.3 (0.05) *
LDL (mmol/L)	2.8 (±0.6)	3.1 (±0.8)	3.1 (±0.4)	0.9 (0.3)	1.4 (0.5)	1.9 (0.3)	0.1 (0.8)
Triglycerides (mmol/L)	1.2 (±0.5)	1.1 (±0.3)	1.2 (±0.4)	0.6 (0.5)	1.1 (0.6)	1.2 (0.8)	0.6 (0.4)
SBP (mmHg)	120 (±11)	112 (±9)	119 (±10)	0.5 (0.5)	0.5 (0.8)	0.2 (0.8)	1.4 (0.5)
DBP (mmHg)	72 (±8)	69 (±6)	70 (±9)	0.2 (0.5)	0.2 (0.8)	0.7 (0.8)	0.8 (0.8)

There was a significant difference in BMI, blood cholesterol, HDL, LDL and triglycerides among the *PRDM16/rs2651899* genotypes groups (*p* < 0.0001, *p* = 0.003, *p* = 0.008, *p* = 0.02 and *p* = 0.003, respectively). There was significance only in HDL among the *PDE4D/rs295978* genotypes groups (*p* = 0.03). ANOVA = analysis of variance, HSD = honest significant difference, MN (±SD) = Mean (±standard deviation), BMI = body mass index, HDL = high density lipoprotein cholesterol, LDL = low density lipoprotein cholesterol, SBP = systolic blood pressure, DBP = diastolic blood pressure. * Statistically significant at *p* value < 0.05.
